# Expression of topoisomerase III **α** in normal and neoplastic tissues determined by immunohistochemistry using novel monoclonal antibody

**DOI:** 10.1054/bjoc.2000.1293

**Published:** 2000-07-24

**Authors:** A J Lodge, J J Anderson, S W Ng, F Fenwick, M Steward, B Haugk, C H W Horne, B Angus

**Affiliations:** Department of Pathology, Department of Virology, University of Newcastle, Newcastle-Upon-Tyne, NE2 4HH; Lab of Gynecologic Oncology, Brigham and Women’s Hospital, Boston, MA 02115, USA; Novocastra Laboratories Ltd, Balliol Business Park West, Benton Lane, Longbenton, Newcastle-upon-Tyne, NE12 8EW

**Keywords:** topoisomerase IIIα, monoclonal antibody, expression, immunohistochemistry, tissues

## Abstract

Topoisomerases are nuclear enzymes that modulate the topological structure of DNA in order to facilitate cellular events such as replication and transcription. These enzymes are also the cellular targets of certain classes of chemotherapeutic agents termed topoisomerase poisons. A new human topoisomerase isoform, IIIa, was discovered in 1996, which is thought to have roles in genome stability and possibly chromosome separation during mitosis. It is possible that novel or existing anti-topoisomerase agents target topoisomerase IIIa, suggesting that this enzyme may have potential as a prognostic marker and chemotherapeutic target. In order to study expression patterns of topoisomerase IIIa we have produced a novel monoclonal antibody to human topoisomerase IIIa (TOPO3a-1A4), and used it to assess topoisomerase IIIa expression in a wide range of normal and neoplastic tissues. We have found that topoisomerase IIIa is expressed in a wide range of tissue types, with especially high concentrations in endothelial cells and stromal fibroblasts. No general relationship was observed between expression of topoisomerase IIIa and proliferation. Expression in neoplastic tissues often paralleled their normal counterparts, although certain tumours showed either increased (e.g. colonic adenoma) or reduced (e.g. gastric carcinoma, small cell carcinoma of bronchus) expression. If topoisomerase IIIa is found to be a target for chemotherapeutic agents, clinical response in different tumour types may be related to topoisomerase IIIa expression, which may be assessed using TOPO3a-1A4. © 2000 Cancer Research Campaign


*British Journal of Cancer* (2000) **83**(4), 498–505
© 2000 Cancer Research Campaign
doi: 10.1054/ bjoc.2000.1293, available online at http://www.idealibrary.com on IDEAL®

### Expression of topoisomerase IIIα in normal and neoplastic tissues determined by immunohistochemistry using a novel monoclonal antibody

**AJ Lodge[1], JJ Anderson[1], SW Ng[3], F Fenwick[2], M Steward[4], B Haugk[1], CHW Horne[4] and B Angus[1]**

[1]Department of Pathology, [2]Department of Virology, University of Newcastle, Newcastle-Upon-Tyne, NE2 4HH; [3]Lab of Gynecologic Oncology, Brigham and Women's Hospital, Boston, MA 02115, USA; [4]Novocastra Laboratories Ltd, Balliol Business Park West, Benton Lane, Longbenton, Newcastle-upon-Tyne, NE12 8EW

**Summary** Topoisomerases are nuclear enzymes that modulate the topological structure of DNA in order to facilitate cellular events such as replication and transcription. These enzymes are also the cellular targets of certain classes of chemotherapeutic agents termed topoisomerase poisons. A new human topoisomerase isoform, IIIa, was discovered in 1996, which is thought to have roles in genome stability and possibly chromosome separation during mitosis. It is possible that novel or existing anti-topoisomerase agents target topoisomerase IIIa, suggesting that this enzyme may have potential as a prognostic marker and chemotherapeutic target. In order to study expression patterns of topoisomerase IIIa we have produced a novel monoclonal antibody to human topoisomerase IIIa (TOPO3a-1A4), and used it to assess topoisomerase IIIa expression in a wide range of normal and neoplastic tissues. We have found that topoisomerase IIIa is expressed in a wide range of tissue types, with especially high concentrations in endothelial cells and stromal fibroblasts. No general relationship was observed between expression of topoisomerase IIIa and proliferation. Expression in neoplastic tissues often paralleled their normal counterparts, although certain tumours showed either increased (e.g. colonic adenoma) or reduced (e.g. gastric carcinoma, small cell carcinoma of bronchus) expression. If topoisomerase IIIa is found to be a target for chemotherapeutic agents, clinical response in different tumour types may be related to topoisomerase IIIa expression, which may be assessed using TOPO3a-1A4. © 2000 Cancer Research Campaign

**Keywords**: topoisomerase IIIα; monoclonal antibody; expression; immunohistochemistry; tissues

Topoisomerases are enzymes that modulate the topological structure of DNA in order to facilitate cellular events such as replication and transcription. This typically involves processes such as supercoil relaxation or DNA unknotting and decatenation. Eukaryotes possess two distinct classes of these enzymes, types I and II. Type I enzymes function by cleaving a single DNA strand, passing the other strand through the gap, and then resealing the break. Type II enzymes function similarly, but cleave both DNA strands, and pass through an entire helix. For a review see Wang (1996).

DNA topoisomerases are known to be the targets of several drugs widely used in cancer chemotherapy. Such drugs act as cellular poisons by binding to, and trapping the DNA-enzyme intermediate 'cleavage complex' on the DNA whilst one or more DNA strands are broken. This leads to DNA fragmentation and ultimately cell death, the likelihood of which is proportional to the level of active enzyme. Hence such agents are often more effective against proliferating cells.

Topoisomerase poisons are known to be type-specific – i.e. they will target either type I or type II enzymes. Agents known to target human topoisomerase I include camptothecin, and its related family of compounds, including topotecan and irinotecan which are used in chemotherapy for ovarian and colorectal cancers (Kudelka et al, 1996; Rothenberg et al, 1996). Agents known to target topoisomerase IIα and IIβ include etoposide, teniposide, daunorubicin and doxorubicin, which are used in therapy of various leukaemias, lymphomas and breast cancer (Fisher et al, 1993; Devine and Larson, 1994; Harris et al, 1997).

The recently discovered human topoisomerase IIIα and β (type I) enzymes represent further possible targets for topoisomerase poisons. The gene encoding topoisomerase IIIα was discovered by Hanai et al who localized it to chromosome 17p11.2–12 (Hanai et al, 1996). The gene encoding topoisomerase IIIβ was discovered in 1997 as part of the immunoglobulin λ gene locus at 22q11 (Kawasaki et al, 1997). Ng et al later discovered that there were multiple transcripts of this gene (Ng et al, 1999); however very little else is known about topoisomerase IIIβ.

As yet, no investigations have been reported concerning the potential of topoisomerase IIIα or IIIβ as targets for anti-neoplastic agents. Indeed little data is available regarding the localization and expression of either isoform. Topoisomerase IIIα has been found to be essential in early embryogenesis in mice, where top3$\alpha^{-/-}$ embryos were found to be non-viable (Li & Wang, 1998). Based on Northern analysis of topoisomerase IIIα mRNA, it is suspected that enzyme is present in many different cell types

*Received 4 August 1999*
*Revised 20 March 2000*
*Accepted 10 April 2000*

*Correspondence to:* AJ Lodge

(Fritz et al, 1997). Sequence tags have been obtained from cDNA libraries derived from adult human heart (Waye et al, 1995) and fetal spleen/liver, and cDNAs have been obtained from hepatoma and T-cells (Hanai et al, 1996). It also seems that expression may be differentially regulated in tissues such as testis (Seki et al, 1998) and also in a developmental manner as the enzyme has been found to be essential in early embryogenesis (Li & Wang, 1998).

In order to study the precise cellular localization and tissue distribution of human topoisomerase IIIα, we have produced the monoclonal antibody TOPO3α-1A4 which can be used to detect this enzyme in formalin-fixed paraffin-embedded tissue sections and Western blots. We have assessed topoisomerase IIIα expression in a wide variety of tissue types, both normal and neoplastic, and here we report our findings.

### MATERIALS AND METHODS

### Production of topoisomerase IIIα recombinant protein

After sequence analysis and comparison with other topoisomerases and proteins, it was decided to generate recombinant protein corresponding to the C-terminal of the molecule. Specific topoisomerase IIIα primers were designed and manufactured using the phosphoramidite method. Total cellular RNA was extracted from a HeLa cell line according to a modified method of Chomczynski and Sacchi (Chomczynski and Sacchi, 1987) and was used as a template for reverse transcription using the antisense primer (5′GCTCGAGTCATCTGTTCTGAGGACAAAAGGG-3′). The resulting reaction mixture served as a substrate for 30 rounds of the polymerase chain reaction (PCR) using the sense topoisomerase III primer (5′-GCTCGAGATGGACAACAGC-CAGCACCCC-3′). The amplification of a 685 bp product was confirmed by agarose gel electrophoresis prior to ligation into pUC57/T (MBI Fermentas). Constructs were transformed into Novablue *E. coli*, and transformants containing appropriately sized inserts were identified by restriction digestion. Topoisomerase IIIα-containing pUC constructs were expanded and the insert excised and sub-cloned into appropriately CIP (calf-intestinal phosphatase)-treated pET 15B expression vector (Novagen). These subclones were re-transformed into T7 polymerase$^{(-)}$ Novablue cells, and transformants were expanded and correctly orientated by restriction mapping. Selected constructs were then re-transformed into T7 polymerase$^{(+)}$ BL21 *E. coli*. Suspension cultures were then grown from colonies from selection plates and protein expression induced by the addition of IPTG (isopropyl thiogalactoside). Induced protein was found to be expressed in the cytosol, with an approximate molecular weight of 25KDa upon analysis by SDS-PAGE electrophoresis. The poly-histidine-tagged protein was then purified by $Ni^{2+}$ chelation column chromatography.

### Hybridoma production

Recombinant protein was injected into five female Balb/c mice in a series of 4 injections given at 14 day intervals. The first consisted of 20 μg protein per mouse in Freunds complete adjuvant, injected subcutaneously. The second was identical but in Freunds incomplete adjuvant. Third and fourth injections were in PBS and administered intraperitoneally. Mice were intravenously boosted with 60 μg of protein four days prior to fusion.

The spleen cells were fused with NS-1/Ag3 myeloma cells using polyethylene glycol, and plated out into HAT-selective growth medium. After 10 days, supernatants from each well were assayed by enzyme-linked immunosorbent assay (ELISA) and colonies showing reactivity were also tested by immunohistochemistry. Specific antibody-secreting hybridomas were picked and transferred to separate wells in a 24-well growth plate. After a period of growth, cells were frozen down, and the supernatants from these colonies screened by Western blotting. Positive lines were subsequently cloned by double dilution, and re-assayed by immunohistochemistry, before being subjected to a further three rounds of cloning.

### Production of a topoisomerase IIIβ expression construct

Recombinant DNA corresponding to human topoisomerase IIIβ was generated by PCR using the primers 3′-AAGGAATTCAT-GAAGACTGTGCTCATGGTT-5′ and 3′-CTCGGATCCTCAT-CATACAAAGTAGGCGGC-5′. This PCR product was then cloned into the yeast expression vector pEG202 in frame with a Lex-A moiety at *EcoRI* and *BamHI* sites. This construct was then transformed into yeast strain EGY48.

### Western blotting

Cell suspensions were solubilized in 2× Laemmli sample buffer (Laemmli, 1970) prior to boiling for 4 minutes. Lysate proteins were then resolved by discontinuous SDS-PAGE using a 10%-T, 2.6%-C acrylamide/bis-acrylamide gel, before being transferred to Hybond C nitrocellulose paper (Amersham) using semi-dry Western blot apparatus. The nitrocellulose was then blocked using 3% BSA made up in PBS-tween buffer (140 mM NaCl, 0.27 mM KCl, 10 mM $Na_2HPO_4$, 1.8 mM $NaH_2PO_4 \cdot H_2O$, 0.05% tween 20), and subsequently incubated with TOPO3α-1A4 (1:100) or anti-Lex A (Invitrogen) (1:5000) in PBS-tween for 90 minutes. After washing, primary antibodies were detected using either peroxide-conjugated rabbit anti-mouse antibody (DAKO) and one-component peroxidase substrate solution (KPL), or by using the 'SuperSignal' kit (Pierce).

### Immunohistochemistry and scoring

TOPO3α-1A4 was tested on paraffin-embedded sections using an indirect strept-ABC method. Briefly, sections were de-waxed and hydrated prior to blocking of endogenous peroxide using methanol/hydrogen peroxide. Antigen retrieval was carried out at high temperature and pressure using citrate buffer (200 mM citric acid, 500 mM NaOH, pH 6.0). Sections were then washed in 1 × TBS (140 mM NaCl, 50 mM Tris/HCl pH 7.6) and blocked using 20% normal rabbit serum (NRS) (in 1 × TBS) for 10 minutes, before incubation with TOPO3α-1A4 at 1:20 (in 20% NRS) for one hour. After washing in TBS, sections were incubated with biotinylated rabbit anti-mouse secondary antibody (DAKO) at 1:500 (in 20% NRS) for 30 minutes. After further washing, streptavidin-biotin (DAKO) was added at 1:100 (in 20% NRS), and the sections incubated for 30 minutes. The final reaction was visualized using 3,3′-diaminobenzidine (Sigma) in a TBS/hydrogen peroxide solution for 3 minutes. Finally, sections were counterstained using Harris' haematoxylin before being blued, and then

© 2000 Cancer Research Campaign dehydrated and mounted in DPX. Tissues were visualized by light microscope, cell types identified, and the proportion and intensity of nuclear staining estimated. Intensity was assessed on a subjective scale of 0 = negative, + = positive, ++ = strong and +++ = very strong staining.

### Tissues

All tissues employed were routinely processed formalin-fixed paraffin-embedded specimens retrieved from the archival files of the Pathology Department, Royal Victoria Infirmary, Newcastle-upon-Tyne, UK.

### RESULTS

### Antibody production

Recombinant protein was generated as described and used to generate an immune response in mice. After the fusion and subsequent assay by ELISA, 120 colonies showed strong reactivity. These supernatants were further screened on paraffin-embedded tonsil sections and 20 colonies were picked and transferred to a fresh 24-well plate. After a period of growth, Western blotting revealed three lines secreting antibody that reacted with proteins of roughly appropriate size. After cloning by double dilution, and re-assay by immunohistochemistry, it was decided that two of these lines showed some non-specific cross-reactivity. In contrast, the remaining line proved highly specific and was subsequently subjected to three rounds of cloning before being successfully re-tested and employed on a panel of normal and pathological tissue sections.

### Antibody characterization by Western blotting

Analysis by Western blot (Figure 1) showed that TOPO3α-1A4 reacted with a protein of around 110 kDa in HT29 (colon adenoma), T47D (breast carcinoma), A431 (epidermal carcinoma) and SKBr3 (breast carcinoma) cell lysates, although there were some faint additional smaller bands present, which have been documented previously (Goulaouic et al, 1999). These may represent products of recently reported alternative transcripts of topoisomerase IIIα (Ng et al, 1999) or degradation products of the topoisomerase IIIα protein. Cell lysates of ZR75 (breast carcinoma), A549 (lung carcinoma) and MDA (breast adenocarcinoma) lines were negative however.

In order to ensure that the antibody did not cross-react with topoisomerase IIIβ, Western blotting experiments were carried out against a lysate of yeast EGY48 cells transfected with the pEG202-topoisomerase IIIβ construct. No reactivity of TOPO3α-1A4 with topoisomerase IIIβ was seen, even though blotting with anti-Lex-A antibody revealed the presence of the topoisomerase IIIβ protein (Figure 2).

### Antibody characterization by immunohistochemistry

Analysis by immunohistochemistry was carried out on a range of neoplastic and non-neoplastic tissues as described below, and summarized in Table 1 and 2. In all tissues showing positive immunoreactivity, cells exhibited a diffuse nuclear stain with dot-like accentuation suggestive of nucleolar localization, most

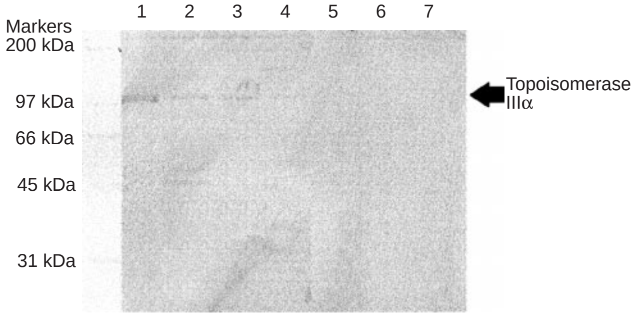

**Figure 1** Western blot showing immunoreactivity of TOPO3α-1A4 in cell lines. Lanes 1–7 represent HT29, T47D, A431, SKBr3, ZR75, A549 and MDA cell lysates respectively. A band of around 110 kDa can clearly be seen in lanes 1–4 (arrow), but expression is not detectable in lanes 5–7

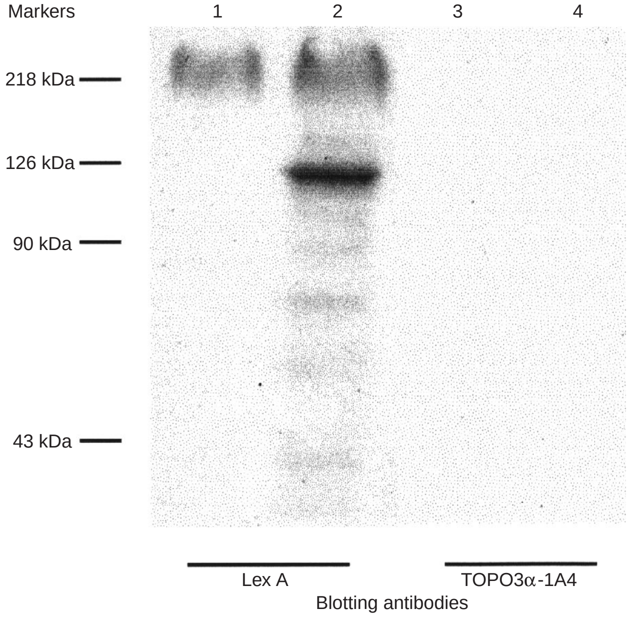

**Figure 2** Western blot showing lack of cross-reactivity with human topoisomerase IIIβ. Lanes 1 and 3 are control yeast lysates (strain EGY48), lanes 2 and 4 are lysates of an EGY48 clone expressing a Lex A-topoIIIβ fusion protein. There is no reactivity of TOPO3α-1A4 with topoisomerase IIIβ, even though blotting with anti-Lex-A antibody reveals the presence of the topoisomerase IIIβ protein evident in cases showing lower levels of nuclear staining. There was no staining of cytoplasm. At all sites, labelling of endothelial cells and fibroblasts was seen. There was no relationship between expression and proliferating compartments with the exception of low grade cervical intra-epithelial neoplasia where the basal layers showed strongest expression (Figure 4F).

### Normal tissues

TOPO3α-1A4 produced moderate to strong immunostaining in epidermis, cutaneous sebaceous glands, transitional epithelium of bladder (Figure 3F), cervical glandular and squamous epithelium, and respiratory epithelium (Figure 3B). In the gastrointestinal tract, staining was observed in gastric pit cells (but not glands), small intestinal glandular and villous epithelial cells, and a proportion of

© 2000 Cancer Research Campaign

**Table 1** Immunostaining for topoisomerase IIIα on a range of normal tissues

| System | Tissue | | Cells | Staining |
|---|---|---|---|---|
| Gastrointestinal system | Pancreas | | Acinar cells | Negative |
| | | | Ducts | 90% ++ |
| | | | Islets | Negative |
| | Stomach (acid secreting mucosa) | | Glandular epithelial cells | Negative |
| | | | Gastric pit epithelial cells | 60%+ |
| | Small intestine | | Glandular epithelial cells | 100% + |
| | | | Villus epithelial cells | 100% + |
| | Colon | | Glandular epithelial cells | 50%+ |
| | Liver | | Hepatocytes | 100% + |
| | | | Bile duct epithelium | Negative |
| Reproductive system | Cervix | | Mucus glandular epithelium | 100% ++ |
| | | | Squamous epithelium | 100% ++ |
| | Endometrium | | Glandular epithelial cells | 100% + |
| | | | Stromal cells | 50% + |
| | Ovary | | Specialised stroma | Negative |
| | | | Oocytes | 100% + |
| | Placenta | | Hofbauer stromal cells | 80% +++ |
| | | | Trophoblast | Negative |
| | Breast | Lobules | Epithelial cells | 50% + |
| | | | Myoepithelial cells | Negative |
| | | Ducts | Epithelial cells | 90% ++ |
| | | | Myoepithelial cells | Negative |
| | Testis | | Germ cells | 50% ++ |
| | | | Sertoli cells | Negative |
| | | | Spermatozoa | Negative |
| Genitourinary system | Kidney | | Tubular epithelial cells | Negative |
| | | | Glomerular cells | 50% ++ |
| | Bladder | | Urothelial cells | 100% +++ |
| Lymphohaemopoietic system | Bone marrow | | Myeloid cells | 10% ++ |
| | Lymph node | | Germinal centre cells | 5% ++ |
| | | | Paracortical cells | 50% + |
| | | | Sinus histiocytes | 90% +++ |
| | | | Endothelial cells | 90% +++ |
| | Tonsil | | Germinal centre cells | Negative |
| | | | Paracortical cells | 10% + |
| | | | Squamous epithelium | 50% ++ |
| Integumentary system | Skin | | Squamous epithelium (all layers) | 100% ++ |
| | | | Sweat gland epithelium | 80% ++ |
| | | | Sebaceous gland epithelium | 100% ++ |
| Endocrine system | Pituitary gland | | Adenohypophysis | 50% + |
| | | | Neurohypophysis | 90% ++ |
| | Parathyroid gland | | All cells | Negative |
| | Thyroid gland | | Follicular epithelial cells | 100% ++ |
| | Adrenal gland | | Cortical cells | 50% + |
| | | | Medullary cells | Negative |
| Nervous system (central) | Brain (cerebral cortex) | | Neurones | 100% ++ |
| | | | Astrocytes | 100% ++ |
| | | | Oligodendrocytes | 100% + |
| Nervous system (peripheral) | Nerve | | Schwann cells | 1% ++ |
| | | | Ganglion cells | 100% ++ |

glandular cells in the large intestine. Pancreatic acinar and duct cells were positive. Amongst endocrine tissues, labelling was observed in epithelial cells of thyroid and parathyroid glands, but not in pancreatic islet cells, and in the adrenal cortex, but not medulla. In breast tissue, weak staining was seen in lobules (Figure 3A) and ducts where a proportion of epithelial but not myoepithelial cells labelled. In the liver most hepatocytes showed weak nucleolar labelling but bile ducts were negative. In the kidney, tubules were negative but around 50% of cells in glomeruli were positive. In bone marrow around 10% of cells showed staining but it was not possible to determine the lineage involved. In lymph node and tonsil, staining was observed in most endothelial cells and sinus histiocytes (Figure 3D); a proportion of paracortical cells stained weakly but in the germinal centres only occasional cells labelled, most likely follicular dendritic cells. In the central nervous system neurons and astrocytes were positive, whilst oligodendrocytes showed slightly weaker labelling. Peripheral autonomic ganglion cells showed strong staining (Figure 3C), and a small proportion of the Schwann cells of peripheral nerve labelled. Ovarian specialized cortical stroma was negative; oocytes were positive. Hofbauer cells of chorionic villi were strongly positive in contrast to the trophoblast which was negative (Figure 3E). Testicular germ cells showed staining whereas sertoli cells and spermatozoa were negative.

© 2000 Cancer Research Campaign 

**Table 2** Immunostaining for topoisomerase IIIα on a range of neoplastic tissues

| System | Tissue | Cells | Staining |
|---|---|---|---|
| Gastrointestinal system | Pancreas | Islet cell adenoma | Negative |
| | Stomach | Adenocarcinoma | Negative |
| | Oesophagus | Squamous cell carcinoma. | 10% ++ |
| | Colon | Adenoma | 100% ++ |
| | | Adenocarcinoma | 10% + |
| Reproductive system | Cervix | Cin I | 50% ++ |
| | | Cin II | 50% +++ |
| | | Cin III | 80% + |
| | | Squamous cell carcinoma. | 80% ++ |
| | Endometrium | Adenocarcinoma | 90% ++ |
| | Ovary | Cystadeno carcinoma | 90% ++ |
| | Breast | Fibroadenoma | 95% ++ |
| | | Adenoma | 60% ++ |
| | | Papilloma | 10% + |
| | | DCIS large cell | 50% + |
| | | ADH | 90% ++ |
| | | Carcinoma | 1% + |
| | Testis | Seminoma | 50% + |
| Genitourinary system | Kidney | Renal cell carcinoma | 10% + |
| | Bladder | Transitional cell carcinoma. | 95% +++ |
| Lymphohaemopoietic system | Bone marrow | ALL | 5% + |
| | Lymphoma | Diffuse large B-cell | 1% + |
| Integumentary system | Skin | Basal cell carcinoma | 60% + |
| | | Squamous cell carcinoma. | 90% +++ |
| | | Malignant melanoma | 90% ++ |
| Endocrine system | Thyroid gland | Adenoma | 50% ++ |
| | | Follicular carcinoma | 50% + |
| | | Papillary carcinoma | 90% ++ |
| | Parathyroid gland | Adenoma | 10% + |
| | Adrenal gland | Phaeochromocytoma | Negative |
| Nervous system (central) | Brain | Astrocytoma | 10% + |
| Nervous system (peripheral) | Nerve | Schwannoma | 90% ++ |
| | | Neurofibroma | 50% ++ |
| Respiratory system | Lung | Squamous cell carcinoma. | 95% + |
| | | Small cell carcinoma | Negative |

### Neoplastic tissues

Considering neoplastic conditions, tumours derived from tissues normally not showing detectable staining were also negative (islet cell tumour, phaeochromocytoma) although a small proportion of cells in a renal carcinoma were labelled (normal renal tubules were negative). Benign tumours (breast, colon (Figure 4A), thyroid (Figure 4E), parathyroid, and nerve sheath) showed expression similar to or greater than their normal counterpart normal tissue (squamous carcinoma of cervix (Figure 4F), non-melanoma skin cancer, seminoma, cystadenoma of ovary, papillary carcinoma of thyroid, endometrial carcinoma). However, some other malignant tumours (breast carcinoma (Figure 4C), follicular carcinoma of thyroid, gastric carcinoma (Figure 4D), small cell carcinoma of bronchus) showed reduced or absent expression. Adenocarcinoma of colon (Figure 4B) showed expression similar to that of normal colonic epithelium, but less than that seen in adenoma. Thus invasive malignant lesions showed variable expression, either greater or less than the tissue of origin. The in-situ neoplasms studied showed expression similar to that of the epithelium of origin (cervical intra-epithelial neoplasia, ductal carcinoma in-situ of breast).

### DISCUSSION

So far very little information is available regarding expression and tissue distribution of human topoisomerase IIIα. Our results have confirmed the suspected wide tissue distribution of the enzyme; we have shown that the enzyme is expressed in a number of cell types, and shows the expected nuclear localization. Expression is also prominent in nucleoli, indicating a possible role in the modulation of the ribosomal genes. We noticed high concentrations of the enzyme in endothelial and stromal cells of all tissues studied.

Unlike topoisomerase IIα, no relationship was observed between expression of topoisomerase IIIα and proliferation. Although widely distributed, a number of cell types in certain tissues showed no detectable staining by both immunohistochemistry and Western blotting. Regarding the former, since endothelial cells and stromal cells were always stained, this observation cannot be attributed to non-immunoreactive (e.g. over-fixed) tissue blocks. However it is possible that topoisomerase IIIα expression is present at a level not detectable by the techniques utilized. No obvious pattern has emerged to explain the finding of reduced or absent labelling in some cells and tissues. Considering neoplastic tissues, it was generally observed that topoisomerase IIIα expression paralleled that of the tissue of origin, for example adrenal medulla and phaeochromocytoma were both negative; thyroid follicular epithelium and carcinoma were both positive. However in some cases, expression was somewhat greater in neoplastic tissues as compared to the tissue of origin; for example colonic adenoma showed greater expression than normal colonic epithelium. Of particular interest is the observation of reduced

 *© 2000 Cancer Research Campaign*

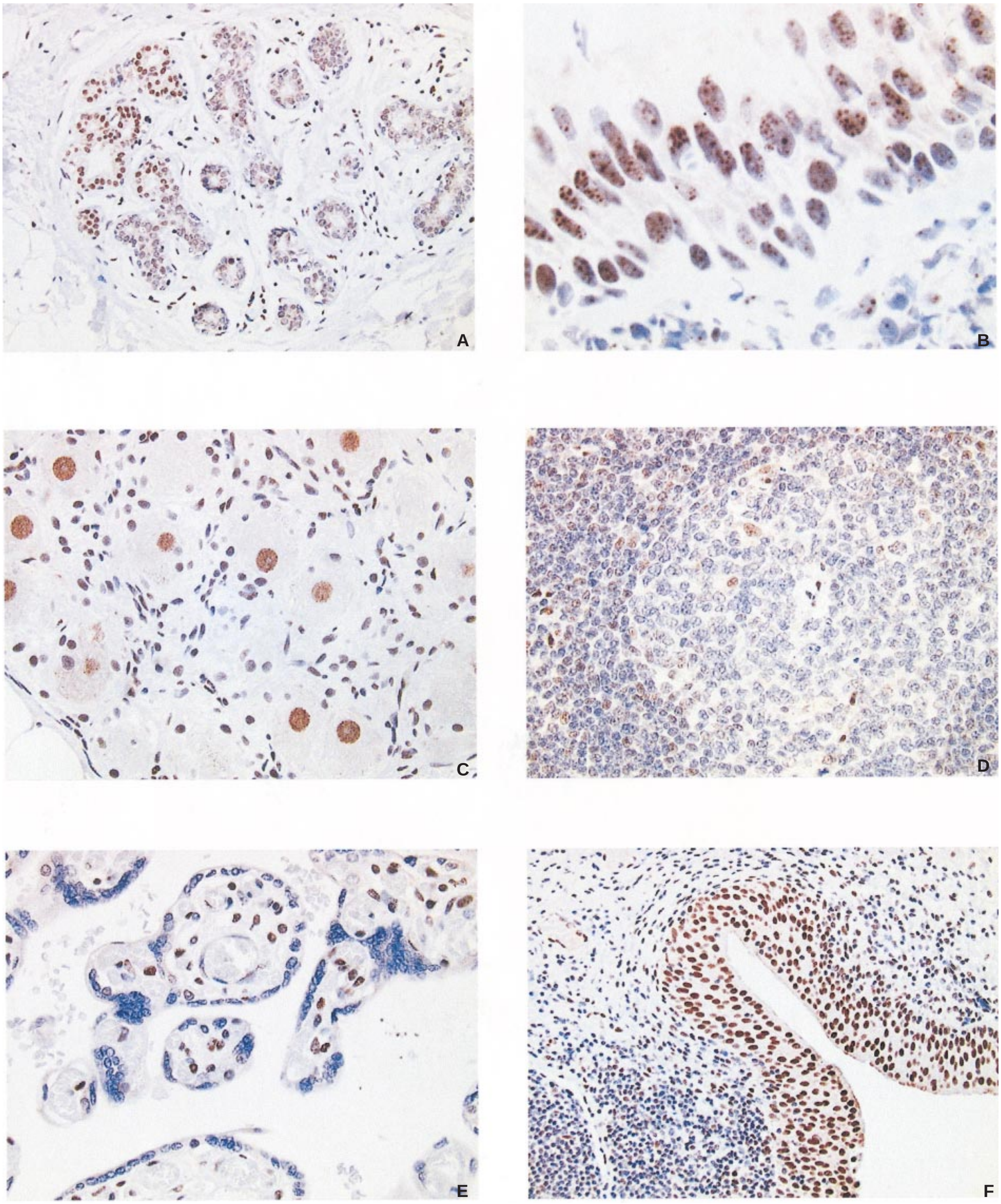

**Figure 3** Immunostaining of normal tissues with TOPO3$\alpha$-1A4. (**A**) Breast lobule; a proportion of acinar cells show staining. (**B**) Bronchus, pseudo-stratified columnar epithelium; nuclei show nucleolar and diffuse labelling. (**C**) Peripheral autonomic ganglion; note intense staining of ganglion cell nuclei. (**D**) Lymph node, germinal centre; most cells are negative; occasional positive cells may represent follicular dendritic cells; the surrounding paracortex shows weak labelling of around half the cells. (**E**) Placenta, chorionic villi; Hofbauer cells within the villi show strong staining in contrast to complete absence in trophoblast. (**F**) Bladder, transitional epithelium; note intense staining of all epithelial cells, contrasting with only a few lymphocytes in the submucosa

(breast carcinoma, follicular carcinoma of thyroid) or absent (gastric carcinoma, small cell carcinoma of bronchus) expression in some malignant neoplasms. Since only a single tumour was studied in each case, further work is required to determine the frequency and significance of this finding.

Although very little information exists regarding the possible function of topoisomerase III$\alpha$ in cell metabolism, there is some evidence linking the enzyme with a role in genome stability, which is consistent with our demonstration of the wide tissue distribution of the enzyme. In yeast, topoisomerase

*© 2000 Cancer Research Campaign* 

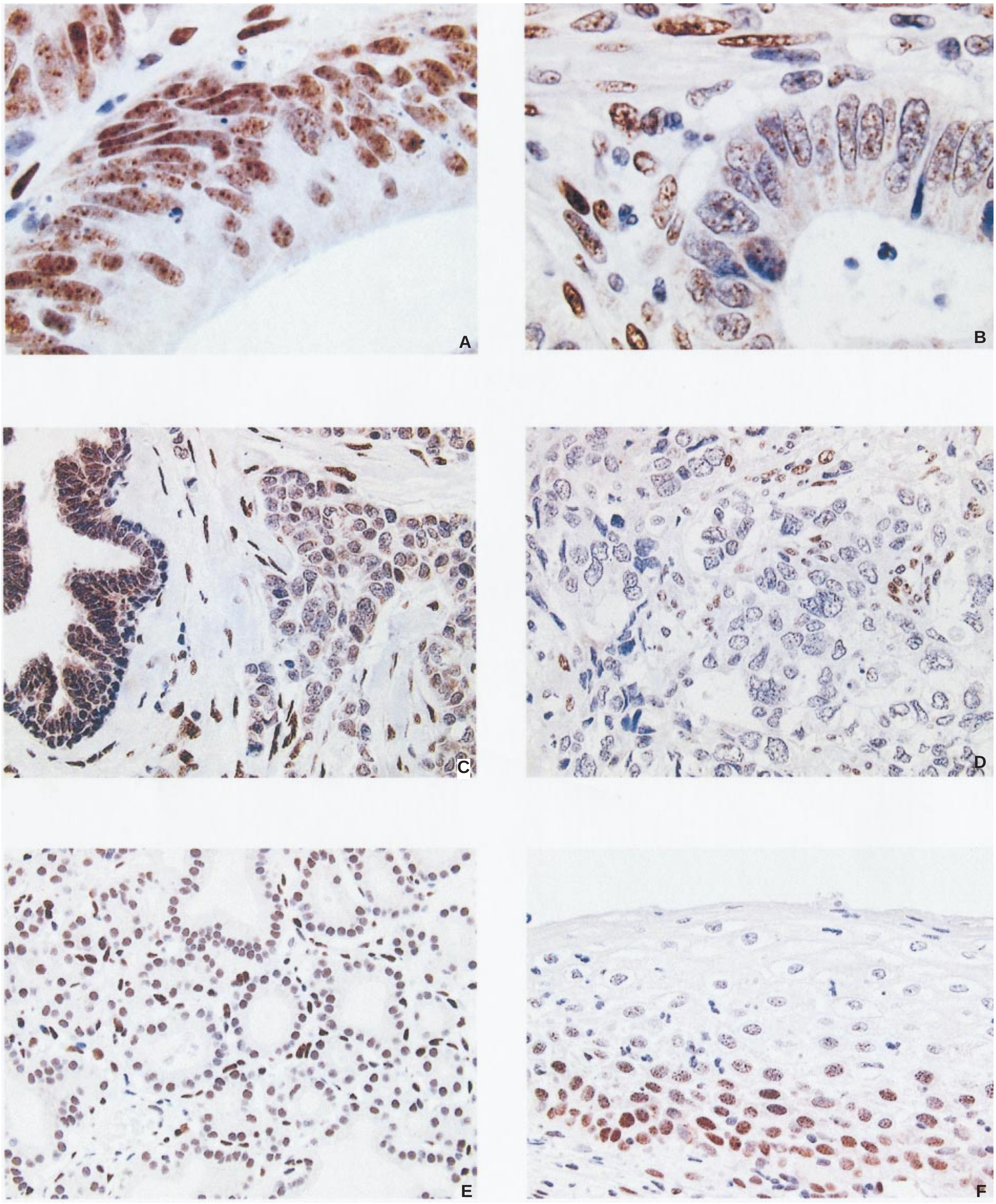

**Figure 4** Immunostaining of neoplastic tissues with TOPO3α-1A4. (**A**) Colon adenoma; note strong nucleolar and diffuse nuclear labelling of epithelial cells. (**B**) Colon carcinoma; note reduced expression compared to the adenoma, and contrasting with strong staining of stromal fibroblasts. The level of labelling was comparable to normal colonic epithelium. (**C**) Breast carcinoma; the tumour shows almost complete absence of detectable labelling, compared to staining of cells in the normal duct shown on the left. (**D**) Gastric carcinoma; no evidence of tumour cell labelling, but stromal cells are positive. (**E**) Follicular adenoma of thyroid; staining is comparable to normal tissue. (**F**) Cervical intra-epithelial neoplasia, grade I; note maximal labelling of cells in the basal layers

III mutations result in cells displaying a slow-growth, hyper-recombinant phenotype. It has been suggested that the former may occur as the result of a potential role of topoisomerase III in DNA replication, where, in addition to topoisomerase IIα, the enzyme would separate replicated chromosomes (Gangloff et al, 1994).

*© 2000 Cancer Research Campaign*

In yeast, topoisomerase III activity is thought to be closely associated with Sgs1, a DNA helicase that also plays a role in genome stability. It has been suggested that the two may form a complex, whereby the function of Sgs1 is to melt the DNA duplex, providing a single-stranded substrate for topoisomerase III. Sgs1 is crucial for maintaining genome stability in yeast, with defective or deficient strains showing hyper-recombination and slow growth. Furthermore Sgs1 is a member of the bacterial recQ family of helicases which also includes the human homologues BRM and WRN. Mutations in these genes are responsible for the human conditions Blooms and Werners syndromes, which display a hyper-recombinant phenotype, with an increased risk of malignancy.

The interaction between topoisomerase III and Sgs1 in yeast has been reported (Gangloff et al, 1994), but the precise biochemistry of their association remains unclear. It seems that in cells with mutant topoisomerase III, a mutation in Sgs1 can actually suppress the slow growth/hyper-recombinant phenotype associated with this defect. In addition, the genetic instability associated with Blooms and Werners syndromes (and also ataxia telangiectasia) can be at least partially alleviated by a mutation in human topoisomerase IIIα (Fritz et al, 1997; Yamagata et al, 1998). It seems possible that a single mutation in either helicase or topoisomerase results in cellular defects, whilst mutations in both genes is less harmful, implying a kind of dominant negative relationship.

An interaction between human topoisomerase type IIIα enzymes and the BRM and WRN proteins has not yet been reported, but is strongly suspected. It may be possible to investigate the relationship between these proteins using TOPO3α-1A4 in order to further elucidate mechanisms involved in these diseases.

The specificity of TOPO3α-1A4 has been demonstrated by ELISA, Western blotting, and immunohistochemistry. We have demonstrated wide distribution of the antigen in a variety of neoplastic and non-neoplastic tissues. Topoisomerase IIIα clearly shares ubiquitous expression with topoisomerase I, the other member of the topoisomerase type I family present in humans. Although a number of reagents have been identified which demonstrate anti-topoisomerase I activity, to date little work has been carried out towards identification of topoisomerase IIIα/β inhibitors. This study represents a wide-ranging overview of expression in normal and neoplastic tissues, taking a single example of each. There is some evidence that levels of expression of topoisomerases may predict response to topoisomerase poisons – therefore if new or existing reagents are found to target the topoisomerase IIIα/β enzymes, it will clearly be of importance to expand our observations concerning the variability of expression in certain malignant tumour types, examining larger numbers of cases and subtypes, and this is the goal of ongoing work in our laboratory.

### REFERENCES

Chomczynski P and Sacchi N (1987) Single-step method of RNA isolation by acid guanidinium thiocyanate phenol chloroform extraction. *Anal Biochem* **162**: 156–159

Devine SM and Larson RA (1994) Acute leukemia in adults – recent developments in diagnosis and treatment. *Cancer Journal For Clinicians* **44**: 326–352

Fisher RI, Gaynor ER, Dahlberg S, Oken MM, Grogan TM, Mize EM, Glick JH, Coltman CA and Miller TP (1993) Comparison of a standard regimen (CHOP) with 3 intensive chemotherapy regimens for advanced non-Hodgkins-lymphoma. *N Engl J Med* **328**: 1002–1006

Fritz E, Elsea SH, Patel PI and Meyn MS (1997) Overexpression of a truncated human topoisomerase III partially corrects multiple aspects of the ataxia-telangiectasia phenotype. *Proc Natl Acad Sci USA* **94**: 4538–4542

Goulaouic H, Roulon, T Flamland, O Grondard L, Lavelle F and Riou JF (1999) Purification and characterisation of human topoisomerase IIIα. *Nucl Aci Res* **27**: 2443–2450

Gangloff S, McDonald JP, Bendixen C, Arthur L and Rothstein R (1994) The yeast type I topoisomerase top3 interacts with Sgs1, a DNA helicase homolog – a potential eukaryotic reverse gyrase. *Mol and Cell Biol* **14**: 8391–8398

Hanai R, Caron PR and Wang JC (1996) Human top3 – a single-copy gene encoding DNA topoisomerase-III. *Proc Natl Acad Sci USA* **93**: 3653–3657

Harris J, Morrow M and Norton L (1997) Malignant tumours of the breast. In: *Cancer: Principles and Practice of Oncology* DeVita V, Hellman S and Rosenberg S (eds). Lipcott-Raven.

Kawasaki K, Minoshima S, Nakato E, Shibuya K, Shintani A, Schmeits JL, Wang J and Shimizu N (1997) One-megabase sequence analysis of the human immunoglobulin lambda gene locus. *Gen Rese* **7**: 250–261

Kudelka AP, Tresukosol D, Edwards CL, Freedman RS, Levenback C, Chantarawiroj P, de Leon CG, Kim EE, Madden T, Wallin B, Hord M, Verschraegen C, Raber M and Kavanagh JJ (1996) Phase II study of intravenous topotecan as a 5-day infusion for refractory epithelial ovarian carcinoma. *J Clin Oncol* **14**: 1552–1557

Laemmli UK (1970) Cleavage of structural proteins during the assembly of the head of bacteriophage T4. *Nature* **227**: 680–685

Li W and Wang JC (1998) Mammalian DNA topoisomerase III alpha is essential in early embryogenesis. *Proc Natl Acad Sci USA* **95**: 1010–1013

Ng SW, Liu Y, Hasselblatt KT, Mok SC and Berkowitz RS (1999) A new human topoisomerase III that interacts with Sgs1 protein. *Nucl Acids Res* **27**: 993–1000

Rothenberg ML, Eckardt JR, Kuhn JG, Burris HA, Nelson J, Hilsenbeck SG, Rodriguez GI, Thurman AM, Smith LS, Eckhardt SG, Weiss GR, Elfring GL, Rinaldi DA, Schaaf LJ and VonHoff DD (1996) Phase II trial of irinotecan in patients with progressive or rapidly recurrent colorectal cancer. *J Clin Oncol* **14**: 1128–1135

Seki T, Seki M, Katada T and Enomoto T (1998) Isolation of a cDNA encoding mouse DNA topoisomerase III which is highly expressed at the mRNA level in the testis. *Biochim et Biophys Acta–Gene Struc and Expression* **1396**: 127–131

Wang JC (1996) DNA topoisomerases. *Ann Rev Biochem* **65**: 635–692

Waye MMY, Cheung HKY, Lam WY, Law PTW, Lo ASY, Lui VWY, Luk SCW, Tsui SKW, Tung CKC, Yam NYH, Liew CC and Lee CY (1995) Miami Winter Biotechnology Symposium Proceedings: Advances in gene technology: protein engineering and structural biology, Whelan WJ (ed), Vol. 6. p. 90. Oxford University Press.

Yamagata K, Kato, J, Shimamoto A, Goto M, Furuichi Y and Ikeda H (1998) Bloom's and Werner's syndrome genes suppress hyper-recombination in yeast Sgs1 mutant: Implication for genomic instability in human diseases. *Proc Natl Acad Sci USA* **95**: 8733–8738

© 2000 Cancer Research Campaign
